# Modeling the Double Layer Capacitance Effect in Electrolyte Gated FETs with Gel and Aqueous Electrolytes

**DOI:** 10.3390/mi12121569

**Published:** 2021-12-17

**Authors:** Roslyn S. Massey, Ravi Prakash

**Affiliations:** Department of Electronics Engineering, Carleton University, Ottawa, ON K1S 5B6, Canada; RoslynMassey@cmail.carleton.ca

**Keywords:** double layer capacitance, bio-electrolytes, biosensors, electrolyte-gated FETs, aqueous and gel bio-electrolytes

## Abstract

Potential implementation of bio-gel Electrolyte Double Layer capacitors (bio-gel EDLCs) and electrolyte-gated FET biosensors, two commonly reported configurations of bio-electrolytic electronic devices, requires a robust analysis of their complex internal capacitive behavior. Presently there is neither enough of the parameter extraction literature, nor an effective simulation model to represent the transient behavior of these systems. Our work aims to supplement present transient thin film transistor modelling techniques with the reported parameter extraction method, to accurately model both bio-gel EDLC and the aqueous electrolyte gated FET devices. Our parameter extraction method was tested with capacitors analogous to polymer-electrolyte gated FETs, electrolyte gated Field effect transistor (EGOFET) and Organic Electrolyte Gated Field Effect Transistor (OEGFET) capacitance stacks. Our method predicts the input/output electrical behavior of bio-gel EDLC and EGOFET devices far more accurately than conventional DLC techniques, with less than 5% error. It is also more effective in capturing the characteristic aqueous electrolyte charging behavior and maximum charging capability which are unique to these systems, than the conventional DLC Zubieta and the Two branch models. We believe this significant improvement in device simulation is a pivotal step towards further integration and commercial implementation of organic bio-electrolyte devices. The effective reproduction of the transient response of the OEGFET equivalent system also predicts the transient capacitive effects observed in our previously reported label-free OEGFET biosensor devices. This is the first parameter extraction method specifically designed for electrical parameter-based modelling of organic bio-electrolytic capacitor devices.

## 1. Introduction

Conventional electronic devices rely on rare earth minerals, extreme processing, and harsh chemical agents; challenges which can be solved by organic material-based electronic devices. Current research explores a host of organic devices tailored for unique applications replacing or complementing conventional devices [1,2]. Among these, there are exciting new technologies that incorporate organic electrolytes in their device architecture to leverage their unique chemical and electrical properties. Electrolyte gated FETs (EGFETs) are attractive for their high gate capacitance, fast switching speed, 3-D printability, and large area scalability. Currently EGFETs are being explored for artificial synapses, wearable electronics, and for biomolecule quantification [3,4,5,6,7,8,9].

One driving factor behind the development of these devices is to produce fast, in-situ bio-molecule quantification methods. Examples of Electrolyte-gated organic FET sensors include Nakata et al.’s Ion Sensitive FET sensor for simultaneous sweat pH and skin temperature measurement, our previous work into cortisol quantification using Organic electrolyte Gated FETs (OEGFETs) as biosensors, and Electrolyte Gated Organic FET (EGOFET) biorecognition switches presented by Parkula et al. [5,10,11]. The EGOFET and OEGFET are two distinct organic FETs with liquid electrolytes in the gate dielectric. Non-specific examples of the EGOFET have electrical output characteristics directly correlated to ion concentrations in the electrolyte [4,11,12,13,14,15]. The label-free OEGFET biosensors have immobilized bio-recognition molecules on the gate, which selectively bind to the target biomolecules and form bound complexes [15]. The resultant gate capacitance change produces device electrical output characteristic changes, correlated to the target biomolecule concentration in the gate bio-electrolyte [15].

Key challenges to EGFET research include device stability, susceptibility to environmental effects and limitations to manufacturing and packaging of aqueous electrolyte devices. To solve these issues, alternate forms of electrolytes are implemented, such as ion polymer matrices, ionic gels and polymer electrolytes [16]. An example of this is Von Seggern et al.’s polymer electrolyte gate that demonstrates high ionic conductivity and high capacitance gating [17,18]. These alternate forms have higher stability, enhanced 3-D printability and good electrical performance, but their long-term electrical compatibility and the electronic behavior of these materials has not been characterized [5,16,19].

The sensing mechanism of EGFET biosensors is based on gate capacitance changes, which is voltage, frequency, and time dependent [20]. Understanding and predicting the internal capacitance mechanism is crucial for optimizing and implementing these devices in simulations, and in fully integrated systems such as sensor nodes. The present method for handling capacitance in transistor modelling techniques is to employ a single, constant capacitance term. Marinov et al. employ this tactic to great effect in their compact DC modelling technique for organic thin film transistors (oTFTs) [21]. The technique is adequate and highly effective for oTFTs as the solid dielectric layers have limited internal capacitor interactions. The trouble is that the lumped capacitance term method is also presently applied to specific biosensing application—such as the Ion Selective (ISFET) extended gate model and the EGOFET model for probe top gates—despite the use of electrolyte gates [22,23]. Under an applied potential, electrolytes form distinctly charged layers with extensive interactions that cannot be captured by a single constant term. EGFET devices have limited applicability due to the inability fully capture their device behavior in simulations [24]. The crux is the absence of a non-destructive, simple, broadly applicable method for capturing low ionic strength electrolyte capacitor behavior that can be incorporated to improve EGFET models. At present, all the existing model capacitance relations are based on the classical equivalence model for conventional Double Layer Capacitors (DLCs) [25,26]. We have shown DLC methods to be incapable of capturing the bio-derived polymeric gel EDLC capacitance effects [5].

To address this, the internal capacitances and time dependent properties of the different electrolyte forms must be investigated. We propose a simple capacitance model tailored specifically to low ionic concentration electrolytes that is suitable for predicting the electrical output characteristics of electrolyte capacitors and electrolyte gated device capacitances. Our method is based on the transmission line model, which incorporates the voltage, frequency, and time dependent properties of complex EDLCs with a facile parameter extraction method. In this work, we examined two electrolyte forms, polymeric bio-gel and aqueous electrolyte solutions, in three common device architectures, a bio-gel EDLC, an EGOFET and an OEGFET. To better approximate the bio-electrolyte behavior from an electronic system’s viewpoint, we built bio-gel EDLCs and capacitor stacks analogous to the EGOFET and OEGFET. All three capacitor stacks were analyzed and a process for modelling their capacitance effects was developed. We report a novel parameter extraction method to reproduce the complex organic bio-electrolytic device behavior in integrated circuit systems, demonstrating that our proposed method accurately predicts the transient behavior of the three tested EGFET forms. We compare our simulation model to the established literature DLC models (Zubieta model and Two branch model), proven for ionic liquid and solid state DLCs, but not for low ionic concentration solutions or polymer capacitors. We establish that our circuit model is capable of recreating EDLC characteristics in the bio-gel EDLC and EGOFET equivalent with a maximum 5% error range in repeated tests. Our subsequently tailored method is furthermore able to recreate OEGFET equivalent characteristics with a maximum 3% error range during repeated experimental measurements.

## 2. Materials and Methods

### 2.1. Device Fabrication

#### 2.1.1. Standard Bio-Electrolyte DLC Fabrication

**Figure 1:**
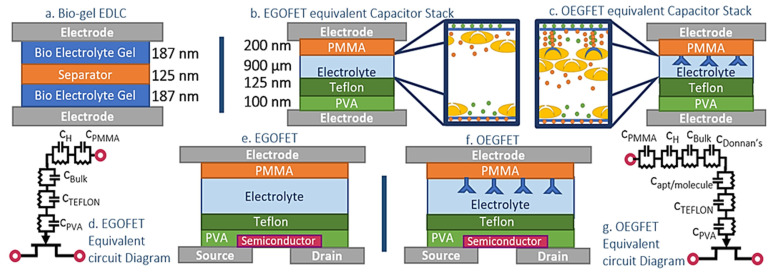
(**a**) Bio-gel EDLC schematic. (**b**) EGOFET equivalent Schematic. (**c**) OEGFET equivalent schematic. (**d**) Equivalent circuit diagram of the EGOFET gate capacitive stack. (**e**) EGOFET device cross section (**f**) OEGFET device cross section (**g**) Equivalent circuit diagram of the OEGFET gate capacitive stack.

Our bio-gel EDLC system is comprised of 5 thin film layers; two composite, polymer-blended bio-electrolyte layers loaded in soft-printed PDMS fixtures, separated by a polyolefin (PPG Teslin^®^ SP600) membrane, and sandwiched between two copper coated glass surface electrodes (Figure 1a) [27].

**Figure 2:**
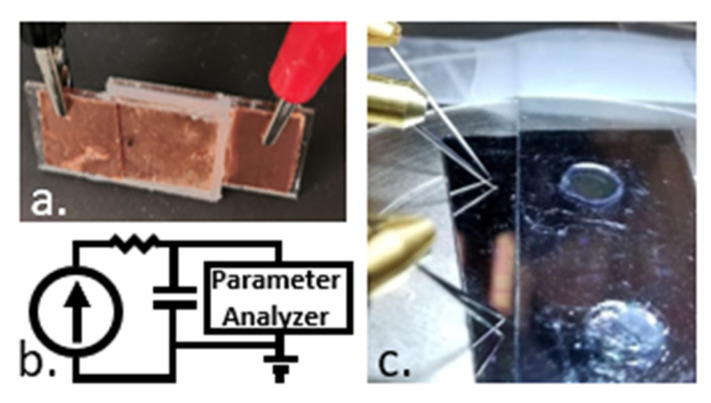
(**a**) Bio-gel EDLC device under test (**b**) Testing setup with constant current source, parameter analyzer and device under test. (**c**) EGOFET equivalent device and OEGFET equivalent device under test.

To fabricate the current collector electrodes, glass slides were coated with adhesive copper tape. Although prone to oxidation, Copper electrodes have been used in the literature to produce ultra-low equivalent series resistance capacitors with high self-resonant frequencies [28]. The 175 µm composite bioelectrolyte is prepared using 1.6 g Carrageenan (Xi’an Lyphar Biotech Co., Ltd., Xi’an City, China), 0.8 g PVA (Sigma Aldrich, St. Louis, MO, USA), 0.1 g Agarose (Sigma Aldrich), mixed in 25 mL of 1% acetic acid solution. A gel is formed by dissolution under constant low temperature (80 °C) while stirring, to form a homogenous solution. The polyolefin separator is coated with Teflon AF 2400 (Dupont, Wilmington, NC, USA) and has a final device thickness of 152 µm, shown in Figure 2a. The final thickness of the separator film was 500 µm. Finally, the devices are sealed to reduce environmental degradation effects (Neutral cure sealant, Dow, Midland, TX, USA). The bio-electrolyte stack thickness was measured to be 400 µm. The capacitor area for the bio-gel EDLC was 2.25 cm^2^.

#### 2.1.2. EGOFET Equivalent and OEGFET Equivalent Capacitor Stack Fabrication

The EGOFET equivalent capacitor stack and the OEGFET equivalent capacitor stacks are two surface structures (Figure 1b,c respectively). A consideration for the EGOFET devices is that in practice, the gate and semiconductor surfaces are often sealed with Bovine Serum Albumin (BSA) to prevent nonspecific adsorption [9,29]. BSA thickness and dielectric permittivity are low, with comparable capacitive behavior to the organic layers used instead for more consistent layer properties whilst still approximating real device operation conditions.

Bottom Surface—The bottom surface is fabricated on a passivated silicon wafer. A 200 nm layer of Chromium is patterned using photolithography and liftoff techniques (in OEGFET devices, this layer would be the patterned electrodes, on top of which would be a semiconductor layer, as seen in Figure 1e,f). A 100 nm thick layer of 2% PVA in water is spun and dried (10 s @300 rpm, 40 s @2000 rpm; bake 10 min @ 95 °C) and a 125 nm layer of Teflon protects the PVA from the aqueous solutions. The bottom surface layer’s purpose is to mimic the protective film over the organic semiconductor of the EGFET devices.

Top gate—A microscope slide is surface activated in a UV ozone chamber at 50 °C for 8 min then spin coated twice with PEDOT:PSS (Sigma Aldrich 739324; 5 s @500 rpm, 40 s @100 rpm; bake 10 min @ 110 °C; 500 nm total), and third layer of a 0.2 mg/mL suspension of PEDOT:PSS and graphene hybrid ink (PEDOT:PSS,G, graphene nanoparticles from Kennedy Labs, Ottawa, ON, Canada) under the same conditions (160 nm thickness). After verifying conductivity, 200 nm of 950 PMMA A4 is coated (8 s @500 rpm, 45 s @4000 rpm) and baked (50 min @ 110 °C). Wells for cortisol aptamer immobilization are crafted by selectively protecting circular areas of the PMMA and spin coating 125 nm of Teflon (AF 2400 × SOL 1% FC40, Dupont, Wilmington, NC, USA; 10 s @500 rpm, 30 s @2500; bake 30 mins @ 160 °C). The multi-layered top gate was then activated using a Samco UV-1 system, with 110 W low-pressure Mercury Lamp at a wavelength of 253.7 nm for 10 min. The exposed PMMA spots became activated, immobilizing drop cast cortisol aptamers to create cortisol specific aptamer wells for the OEGFET equivalent and blank wells (drop cast DI water) for the EGOFET equivalent stack. Soft-printed PDMS device wells were used to control the total electrolyte volume and stack height for the EGOFET equivalent and EGOFET equivalent capacitor stacks.

Electrolyte solutions—The electrolytes used are cortisol solutions in an ascorbic acid (AA) buffer (pH 6.8, conductivity 2.92 mS/cm) of concentrations (from a stock solution of cortisol in AA 50 mg/50 mL, a series of dilutions of 5 ml in 50 mL to produce 6 dilutions from the stock). The cortisol dilutions range from the most concentrated (Cortisol 1) at 5.4 mM, decreasing in concentration by a factor of 10 with each dilution to Cortisol 6 at 54 pM.

The EGFET equivalent capacitor devices are constructed by interposing 10 µL of electrolyte solution between the bottom surface and the PDMS well that encloses the electrolyte and acts as a separator. The separation between the two surfaces was kept consistent at 900 µm using PDMS as separator. The resulting EGFET devices had an area of 0.2 cm^2^.

### 2.2. Device Testing

Constant charge measurements were conducted on the bio-gel EDLCs, EGOFET equivalent and OEGFET equivalent capacitors under low voltage (9 V) and low current (0.1 mA) bias, to mimic the operating conditions of our OEGFET sensor set up [15]. The capacitor, in series with a 10 kΩ resistor, was connected to a Keithley 220 current source that ran 600 s, which was determined as time required for the bio-gel EDLC to reach 0.9 V, 100 mA current pulse (compliance: 10 V) (Figure 2b). The voltage across the capacitor was measured with a HP4156A semiconductor parameter analyzer over a 30-min window. Comparisons of simulated to real data are performed using Root Mean Square (RMS) error calculations.

Impedance testing on all devices was performed to directly capture the AC electrical characteristics. An Agilent 4294 A impedance analyzer unit with Agilent 42941 A probe kit was used to run the tests. All devices were tested over the range of 40–100 kHz to collect impedance, phase angle, capacitance, and resistance data with respect to frequency.

### 2.3. Device Modelling Methods

**Figure 3:**
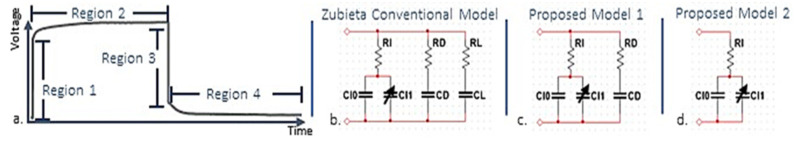
(**a**) Data regions used for parameter extraction shown on a representative curve. (**b**) Zubieta DLC model Equivalent Circuit. (**c**) Proposed Model 1 equivalent circuit implemented with bio-gel EDLC and EGOFET equivalent (**d**) Model 2 equivalent circuit implemented with OEGFET equivalent.

In our previous work, we established that out of the existing DLC models, the Zubieta DLC model was best at approximating bio-gel EDLC behavior. The Zubieta capacitance equivalent circuit (Figure 3b) uses multiple RC branches to predict conventional DLC behaviour and approximate the electric double layer electrochemistry and interfacial tension theory [19,22,23]. However, the conventional Zubieta DLC model fails to fully capture the rise time, maximum voltage, and discharge behaviour of EGFET capacitor devices [5]. In order to better approximate EGFET capacitor behaviour, we propose a novel parameter extraction method and equivalent circuit (Figure 3c). Our equivalent circuit models (proposed model 1; Figure 3c) improve simulation of rise time, maximum voltage and discharge. The OEGFET equivalent data demonstrated differences in the capacitance method, to which we fit the equivalent circuit model 2 as demonstrated in Figure 3d. To demonstrate its efficacy with EGFET capacitors, the equivalent circuit models were implemented in Multisim 14.2 with the extracted parameters and tested under conditions analogous to the charge test. With a 10 kΩ resistor in series, a simulated charge test was performed in Multisim, with a 10-s zero current delay, 600-s 100 mA pulse at 10 V compliance, followed by a 30-min data collection period.

The charging behaviour of the EGFET capacitors features an initial jump in voltage which is due to internal resistances (Figure 3a). In the Zubieta DLC model, the first branch has a low resistance value approximated by the first 20 ms of the capacitive charging. Due to this approach, the Zubieta DLC model is quite inflexible, and has disproportionately variable results with the tested devices. In our parameter extraction method, the first RC branch resistance is approximated by the entire voltage jump. Using Equation (1), the resistance is calculated using the constant current value and the voltage jump in region 1 of Figure 3a.

(1) $$R_{1}=\frac{\Delta V}{I}$$

(2) $$R_{2}=10R_{1}$$

The Zubieta DLC model capacitance extraction expects small voltage changes resulting from resistance effects, which is again highly restrictive towards bio-gel EDLC implementation. The large resistive jump is observed in all of three EDLC devices, preventing the conventional approaches from being applied consistently. Considering this, we preferred a method similar to the Two-branch model for extracting the immediate branch capacitances. The conventional Two-Branch model uses two RC branches to approximate DLC behavior, the first for main charge storage and the second for internal redistribution [30,31]. We applied Equations (3) and (4) to the real collected transient data find the *C_I_*_0_ and *C_I_*_1_ values (extracted using two points in the charging region shown as region 2 in Figure 3a). The Two-Branch DLC model assumes the resistive effects are extremely small compared to the overall charging, which is the inverse of our previous EGFET capacitor charging behavior. As such, voltages at selected points were estimated using the voltage difference in Equation (1) as a reference voltage. Point 1 (at time *t*_1_ and voltage *V*_1_) is positioned at 25% of the voltage increase during the charging portion of region 2, and point 2 (*t*_2_, *V*_2_) at 50%.

(3) $$C_{I0}=\left(\frac{t_{1}}{V_{1}}-\frac{V_{1}\times t_{2}-t_{1}\times V_{2}}{V_{2}^{2}-V_{1}\times V_{2}}\right)\times I_{C}$$

(4) $$C_{I1}=2\times \left(\frac{V_{1}\times t_{2}-t_{1}\times V_{2}}{V_{1}\times V_{2}^{2}-V_{1}^{2}\times V_{2}}\right)\times I_{C}$$

An assumption that is consistent across all the proposed conventional DLC models is that the delayed branch resistance is significantly larger than the immediate branch resistance. This assumption greatly simplifies their extraction steps. Under this assumption, we assign the *R_D_* value to be 10 times the *R_I_* value, as in Equation (2). Region 3 has a similar voltage drop to region 1, which is expected given the stacked nature of the devices. Continuing with the Two-branch DLC model is only feasible until this point, as it relies on conventional capacitor equations which we have demonstrated do not apply to the EGFET capacitor [5]. As the capacitance term for the delayed branch cannot be extracted from equations analogous to the Two-Branch DLC model, we applied equations derived from Equation (5) to calculate the *C_D_* value, as seen in the Zubieta DLC model (Equation (6)). This capacitance term is calculated over region 4.

(5) $$V=\frac{Q}{C}$$

(6) $$C_{D}=\frac{Q}{V}-\left(C_{I0}+\frac{C_{I1}}{2}\times V\right)$$

In addition to revamping the parameter extraction approach as described above, we removed the long-term RC branch as its main contribution is in the long-term discharge which does not have a large impact on our model, the same conclusion reached by Faranda et al. [30]. The final equivalent circuit model looks similar to a hybrid Two-Branch DLC model, with a different approach for extracting the *R_D_* and *C_D_* values that does not rely on resistance or capacitance values captured from conventional capacitor equations [30]. The similarity of our final implementation to the Two-Branch DLC model was unexpected given the Two-Branch’s poor performance in our previous work. However, the reliance on the τ extraction for the second branch likely negatively impacted the overall performance there since the EGFET capacitors do not follow the basic capacitance governing equations linking τ to rise time.

The EGFET sensing mechanism functions as electrolytes with a membrane under DC potential, analogous to a conventional DLC [5,19]. They have a measurable device capacitance dependent voltage represented as the charge build-up *Q* divided by capacitance *C* (5). Equation (7) describes the differential capacitance, the change in charge for a given voltage. This value describes the electrochemistry of EDLs, making it a key parameter for understanding DLC and EGFET operation.

(7) $$C_{diff}\left(V\right)=\frac{dQ}{dV}{|}_{v^{\prime }}$$

The total per unit area gate capacitance of the OEGFET equivalent and EGOFET equivalent, *c_di_* has contributions from each dielectric layer (Figure 1d,g), along with the bio-electrolyte film. The stacks of solid and liquid are distinct, and therefore act as series capacitance terms (Equation (8)). *c_PMMA_*, *c_PVA_* and *c_Teflon_* are calculated directly as their dielectric values are consistent and consistent within the literature.

(8) $$c_{di}={\left(\frac{1}{c_{PMMA}}+\frac{1}{c_{Electrolyte}}+\frac{1}{c_{Teflon}}+\frac{1}{c_{PVA}}\right)}^{-1}$$

EGOFET equivalent electrolyte capacitance is due to the formation of an EDL at the electrode surfaces under applied potential. As demonstrated in our previous work, the capacitance of the EDL in these electrolyte devices can be approximated as capacitive contributions from the electrolyte bulk (*c_Bulk_*), and a diffused Helmholtz double layer (*c_H_*) [19]. The lumped gate dielectric capacitance for EGOFET equivalent is reported in Equation (9).

(9) $$c_{EGOFET\;equivalent}={\left(\frac{1}{c_{PMMA}}+\frac{1}{c_{H}}+\frac{1}{c_{Bulk}}+\frac{1}{c_{Teflon}}+\frac{1}{c_{PVA}}\right)}^{-1}$$

The capacitance mechanism of the OEGFET equivalent (Equation (10)) is governed by the presence of aptamers. They create a region of reduced ionic mobility at the interface of the device due to steric hindrance and form a distinct layer within the electrolyte away from the solid-liquid interface. The bound aptamer-cortisol molecule system acts as a porous, solid dielectric film. The reduction of charged particle movement prevents effective formation of the EDL diffuse layer, further inhibited by localized charge from aptamer chains. This creates a potential difference between the two distinctly charged layers, the bulk electrolyte and that bound by the aptamer complex, called a Donnan’s equilibrium. The capacitance from this charge separation, *c_Donnan’s_* is proportional to ionic concentration of bulk electrolyte.

Due to the concentration gradients across this layer, the chemical potential causes an alignment of biomolecules at the film, which increases in thickness with electrolyte concentration. This film will have an additional pseudo dielectric effect (*c_aptamer_ _complex_*) as balancing the equilibrium created by the biofilm affects the potential experienced across the resulting dielectric stack.

(10) $$c_{OEGFET\;equivalent}={\left(\frac{1}{c_{PMMA}}+\frac{1}{c_{H}}+\frac{1}{c_{Donnan^{\prime }s}}\;+\frac{1}{c_{aptamer\;complex}}+\frac{1}{c_{Bulk}}+\frac{1}{c_{Teflon}}+\frac{1}{c_{PVA}}\right)}^{-1}$$

The difference between the EGOFET equivalent and the OEGFET equivalent hinges on the capacitance mechanism differences. In our proposed OEGFET equivalent circuit model 2, the initial movement of charge is dictated by the formation of the aptamer complex layer; the forced Donnan’s equilibrium and concentration gradient across the aptamer-biomolecule film forms as soon as the aptamers contact the solution. Ergo, device charging in the OEGFET equivalent is fundamentally different from the other Bio-gel EDLC devices, as the other Bio-gel EDLC charging times rely on the slower charge physical movement as well as electrolyte. For this reason, the proposed model for the OEGFET equivalent does not require the delayed RC branch, model 2 as depicted in Figure 3d, as the movement of charge in the electrolyte occurs at surface contact, rather than with applied current. The specific biosensor capacitance modelling literature is limited, making this of key importance.

## 3. Results

### 3.1. Bio-Gel EDLC Simulation

Each bio-gel EDLC charge test was performed a minimum of three times, and the extracted parameter or value was averaged to improve the accuracy of our collected parameters. The tested bio-electrolyte stack produced an extended charging time of 600 s with a τ of 165 s and a maximum rated voltage of 10 V, reaching a maximum charge differential of 1 V. Two quantitative methods were used, comparison of the peak voltages and a RMS error between the real and simulated charging curves. Comparing the repeated charge test curves and the simulated charging curves demonstrated a difference in peak voltage of less than 0.02 V, and an overall difference of 2.5% between the charging curves and simulated data curves. The percent error of 2.5% is within the capacitor’s common tolerance limits.

**Figure 4:**
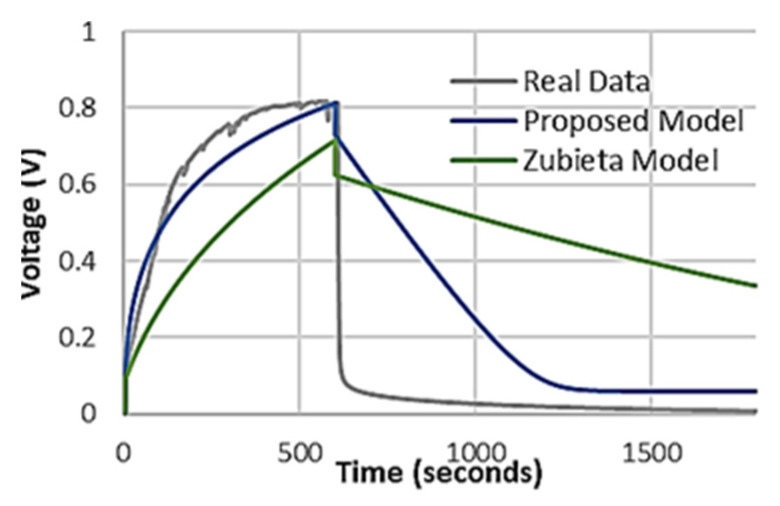
Real charge test Bio-gel EDLC data compared to the simulated Zubieta and Proposed equivalent circuit Model 1.

Both the Zubieta DLC and our proposed equivalent circuit model 1 are plotted against real Bio-gel EDLC data in Figure 4. The key areas in which our model functions significantly better than the Zubieta DLC model are charging behavior, maximum voltage and the discharge characteristics. The real data demonstrates a linear voltage-charging time section, followed by a second section as the capacitor approaches a maximum voltage. Neither the Zubieta nor our proposed simulation method are capable of fully approximating this behavior; however, the proposed circuit model 1 introduces far less error than the Zubieta and reaches a maximum voltage similar to real data. The discharge behavior of the real devices demonstrates a fast discharge. The Zubieta DLC simulation has a voltage drop profile similar to ours, but with a significantly slower discharge. Another crucial limitation of the Zubieta DLC model was that for maximum voltages below 1 V, the relations began to breakdown as seen from the findings where the discharge profile is poorly captured.

The immediate RC branch (the dominant branch for the charging portion of the devices) has a capacitance of 32 F, with a series resistance of 0.94 Ω. These values do not agree with the real Bio-gel EDLC parameters extracted from our measured data; capacitance value as 80 µF, with series resistance of 85 Ω and parallel resistance of 6.4 E−11 Ω [32].

### 3.2. EGOFET Equivalent Capacitor Simulation

The EGOFET equivalent rise time was captured to be 600 s, consistent with the bio-gel EDLC, but with a τ of a few milliseconds. This is a major difference between the bio-gel EDLC, EGOFET equivalent and the literature DLCs. Another significant difference between the bio-gel EDLC and the EGOFET equivalent is the maximum voltage. The Bio-gel EDLC will charge to approximately 1 V, as water begins to dissociate at 1 V [33]. The EGOFET equivalent demonstrated a maximum voltage of up to 11 V. Repeated data collection runs demonstrated a maximum voltage difference of 0.23 V, and less than 3% percent error between repeated real data curves and simulated data, which is well within common variation allowances for capacitors.

**Figure 5:**
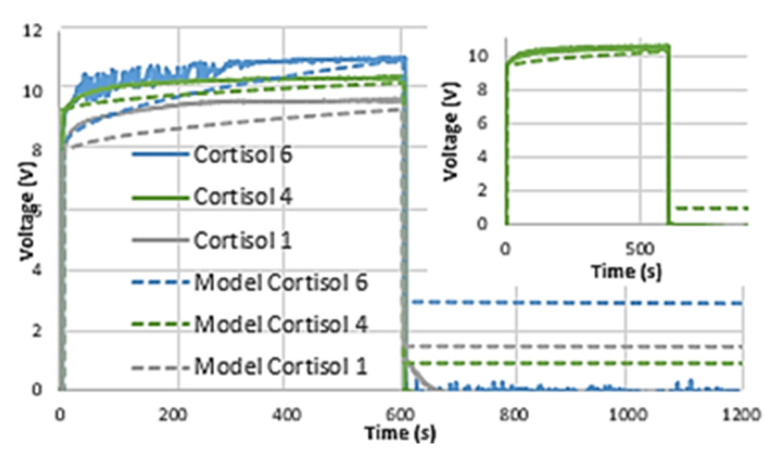
EGOFET equivalent real data compared to equivalent circuit model 1. Inset: Cortisol 4 dilution showing real data to model comparison.

Our proposed equivalent circuit model 1 effectively captures the initial voltage jump and reaches the same maximum voltage, a clear improvement on the previously reported Zubieta DLC comparisons (Figure 5). The discharge is also more effectively modeled than the Zubieta, resulting in significantly less error than the Zubieta DLC method. However, as in case of the bio-gel EDLC, it again demonstrates high capacitances (immediate branch capacitances of 7–95 Farads) that do not equate with measured bulk capacitances of 80 µF.

### 3.3. OEGFET Equivalent Capacitor Mechanism Simulation

**Figure 6:**
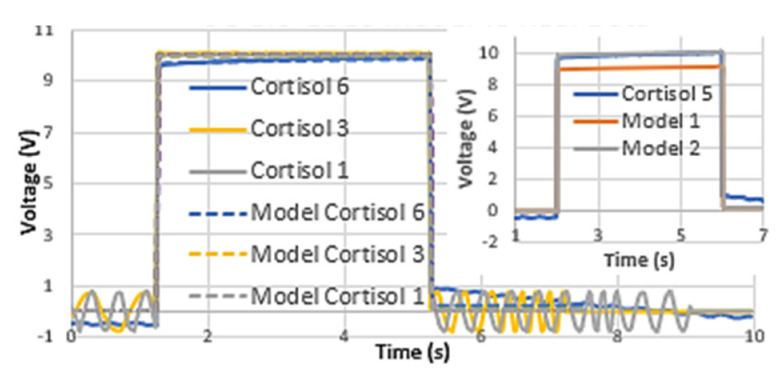
Comparison of real OEGFET equivalent data to equivalent circuit model 2 for all cortisol dilutions. Inset: Comparison of model 1 and model 2 to cortisol dilution 5.

The OEGFET equivalent demonstrated a rise time of a few milliseconds, reaching 98% of the total charging in less than 10 ms. Equivalent circuit Model 1 is not suitable for capturing the OEGFET equivalent performance, as shown in Figure 6. The increase in voltage is less than 1% over the charging time, but the simulation approximates charging to be significant. The proposed equivalent circuit model 2 results in a much more effective capture of the OEGFET equivalent performance, with far better discharge behavior than the Zubieta DLC parameter extraction method. Repeated charging and simulation data had peak voltage differences of 0.22 V, and less than 2% percent error in the charging curves between real and simulated data; well within common capacitor variations.

Similar to the findings for the EGOFET equivalent, the overall capacitances are significantly larger (between 7 and 15 F) than the measured value of 1.3 µF, with lower internal resistances of 85–105 Ω compared to the measured 5 kΩ.

## 4. Discussion

Our proposed EDLC equivalent circuit models demonstrate more effective reproduction of the organic, low ionic strength capacitor’s transient behavior than the conventional DLC models. The difference in the model capacitance parameters compared to the measured capacitance is due to the equivalent circuits’ simplicity and its reliance on simulated circuit components. The measured values are comparable to the literature DLC values, but the disagreement with the extracted parameters indicates that although our circuit model is more capable of approximating the electrical output characteristics of the capacitor than conventional models, it does not approximate the internal interfacial surface chemistry at the gate-electrolyte interface. This is anticipated in such approaches since bio-EDLC capacitors are highly complex, and modelling each of the interactions separately would require a much more cumbersome characterization and device analysis process based on design parameters as well as output characteristics. Therefore, our holistic approach for reproducing the input-output characteristics is desirable as it allows accurate simulations for each of the devices with a few data sets.

The accuracy of our simulations is examined quantitatively through the peak voltage and percent error between simulated and real data. Peak voltage values describe the maximum charge storage capabilities of a capacitor. This is key to simulating device behavior in circuits. In all instances, our proposed models captured with real data peak voltages to within 5%. The charging curve captures the time dependent properties of the internal charge distribution, another significant factor in circuit simulation of electrolyte gated capacitors. Our proposed model demonstrated a maximum of 3% error between the real data and the simulated data, indicating that our models are closely mimicking the charging curves of the real data.

The improved accuracy of the proposed equivalent circuit model simulations demonstrates their accuracy for recreating the transient properties of these devices, without the need for modelling the highly complex internal interactions. As described in this section, our proposed simplified circuit simulation models allow for simple yet accurate predictive transient analysis of such systems without the need for an overly complex extraction procedure.

### 4.1. Performance of Bio-Gel EDLCsimulation with Proposed Equivalent Circuit Model

The Bio-gel EDLC results were consistent with our previous findings that the Zubieta DLC model was not capable of capturing key charge/discharge behavior of aqueous electrolytic capacitors [5]. We have previously demonstrated that the low-ionic strength electrolyte gated capacitors cannot be described by conventional capacitor equations. Therefore, parameter extraction methods—such as the Zubieta model—based on conventional equations introduce significant error into the model.

The Bio-gel EDLC rise time was consistent with conventional DLCs, and significantly higher than our previously reported Bio-gel EDLCs [5,34,35,36]. The literature DLCs demonstrate time constants (τ = RC = 63% Maximum Voltage) in the multiple minutes range [37] with Negruoi et al. reporting a τ of 500 s, a rise time of 750 s, and a fall time of 600 s [38] and Zubieta and Faranda reporting τ values of 100 s and 250 s, respectively [30,39]. The charging time of the bio-gel EDLC is independent of the final capacitance value [40,41] and related to the accumulation of absorbed or structural molecules on the electrodes preventing the dipole-orientation of capacitive molecules. The tested bio-gel EDLC has a more robust gel layer through increased crosslinking of the two composite-biopolymer components. This increased crosslinking produces more local high-density charge areas within the dielectric bulk, interacting with electrolyte molecules and prevents their dipole orientation [42]. As charging time is related to expansion of dipole orientated aggregate domains in the dielectric, the crosslinking interacting with the electrolyte molecules will slow the charging time [40,41]. However, despite the robust gel layer, leakage currents were still observed in the range of 100 nA. The range of leakage current values depended on the charge of the capacitor, increasing with the capacitor charge.

The immediate RC branch (the dominant branch for the charging portion of the devices) is comparable to the literature Zubieta and Two-Branch DLC models, but these values do not agree with the measured Bio-gel EDLC parameters. Despite this, we have achieved a more effective method for capturing this capacitor’s external electronic behavior, which can be implemented to optimize these devices in electronic systems.

### 4.2. Performance of EGOFET Equivalent Capacitor Simulation with Proposed Circuit Model 1

The EGOFET equivalent rise time was consistent with the bio-gel EDLC, but with a τ of a few milliseconds (Figure 5). This is a major difference between the bio-gel EDLC, EGOFET equivalent and the literature DLCs. DLC capacitance depends on solvent dipole charge localization and orientation due to the external field, which takes nanoseconds when it is the sole exerting force [27,31]. Adsorbed water molecules interacting through hydrogen bonding to solvent molecules can force the solvent into lower energy sp3-like bonding structures. The contest between dipole orientation energy and hydrogen-bonded absorption results in capacitance-inducing dipoles distributed in low density, where charging time is influenced by the expansion time of the dipole-oriented domains. The charging time is therefore a function of device geometry and materials selection. Unlike DLCs, the EGOFET equivalent and OEGFET equivalent have smooth electrodes, reducing surface area and therefore adsorbed molecule quantity. In addition, our bulk bio electrolyte is in an ascorbic acid buffer of mild acidic nature which results in higher hydrogen bonding interrupted lifetimes [43]. In addition, the movement of molecules in the electrolyte will increase the charging capacity of these devices. This contributes to the desired faster charging and affects the internal capacitance mechanism.

Another significant difference between the bio-gel EDLC and the EGOFET equivalent is the maximum voltage. The relationship based on Equation (5): *Q_max_ = C × V_max_*, where *Q_max_* is maximum charge stored on a capacitor, and *V_max_* is the breakdown voltage of the dielectric explains the disparity in maximum voltage. The Bio-gel EDLC will charge to approximately 1 V, as water begins to dissociate at 1 V [33]. The EGOFET equivalent demonstrated a maximum voltage of up to 11 V. This was made possible because of the stacked dielectric capacitor form. As the layers are distinct, they act as capacitors in series dropping the voltage applied across the electrolyte layer. This allows for significantly higher maximum voltages applied to the EGOFET equivalent than to the bio-gel EDLC, embodied by the EGOFET equivalent insignificant levels of current (−10 to 10 nA) during the I-V sweeps for leakage.

Our proposed equivalent circuit model 1 effectively captures the initial voltage jump and reaches the same maximum voltage, a clear improvement on the previously reported Zubieta DLC comparisons (Figure 5). The discharge is also more effectively modeled than the Zubieta, resulting in significantly less error than the Zubieta DLC method. The measured capacitance is comparable to the literature DLC capacitor values, but also significantly lower than the extracted parameters [34,35,36]. This again indicates that the circuit model 1 equivalent circuit is most likely matching the output characteristics rather than predicting the internal interactions, an observation that extends to the literature Two-branch DLC models [30]. The Two-Branch DLC model specifically attempts to approximate the electrical characteristics of their devices rather than attempting to approximate internal biochemistry and has been highly effective for DLCs. Thus, accurate electrical behavior simulation does not necessitate accurately predicting internal characteristics [3,26]. Our proposed parameter extraction method reproduces the rise and discharge of the real data with errors of between 0.8–5%, which is well within tolerance limits of regular capacitors.

### 4.3. Performance of OEGFET Equivalent Capacitor Simulation with Proposed Circuit Models

Unlike the other forms of bio-gel EDLC, the OEGFET equivalent demonstrated a rise time of a few milliseconds. This is consistent with the capacitance method proposed in Equation (10), as it demonstrates the absence of ‘charging’ in the capacitors. Charging time is not related to the final device capacitance; long charging times have been observed in low and high capacitance DLCs [30]. Instead, charging time is a function of dipole orientation and movement of charge within the capacitor. The difference with the OEGFET equivalent is that the movement of charge and dipole orientation begins the moment the electrolyte is sandwiched in the gate. As described, the Donnan’s equilibrium produces a charge differential across the aptamer-complex layer which acts as an applied potential to the solvents in the solution leading to the millisecond charging times observed.

Based on Figure 6, our equivalent circuit model 1 is not suitable for capturing the OEGFET equivalent performance. The delay in capacitance is rendered negligible by the presence of Donnan’s equilibrium, as previously established. To reduce these effects, the second RC branch—which is used to approximate delayed capacitance effects—was removed. The proposed equivalent circuit model 2 results in a much more effective capture of the OEGFET equivalent performance, with far better discharge behavior than the Zubieta DLC parameter extraction method. We furthermore predicted the rise and discharge characteristics in the real data with errors of less than 3%, which is well within tolerance limits of regular capacitors. This investigation also supports our previous findings for OEGFET cortisol biosensors, that the presence of the aptamer layer results in a rapid response to cortisol concentration, as corroborated by circuit model 2.

**Figure 7:**
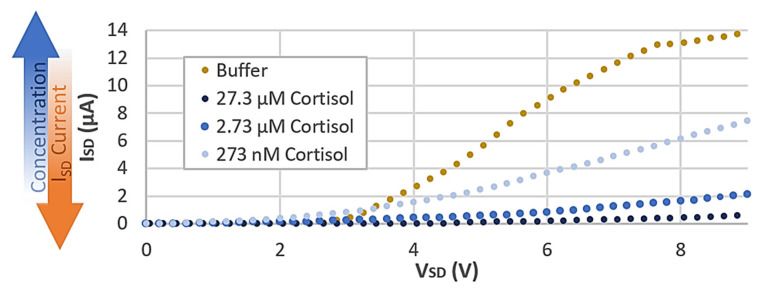
Reproduced previously published data [5] showing increasing output current under the same conditions for decreasing cortisol concentration solutions.

When incorporated into a cortisol biosensor device, concentration changes in the capacitive layer induce observable changes in I-V characteristic curves as shown in our previously published data (Figure 7). Current-Voltage sweeps were performed with the semiconductor parameter analyzer. The sweeps were performed over the range of 0–15 V, with a current compliance of 0.1 A transmitted to the test capacitor and the current data collected to analyze leakage across the capacitor. These results are independent of transient properties which are consistent across all concentrations (Figure 6). All TFT devices exhibit increasing current past saturation—clearly observed in the higher concentrations that reach saturation quickly—with output current increasing linearly with voltage. This is often attributed to charge injection. In our tests, the OEGFET equivalent stack sustains high charge injection under the high applied current bias, thus producing matching voltage levels as expected by the standard capacitance mechanism. Low leakage currents (less than 8 nA) indicate no dielectric breakdown despite achieving voltage maximums around 10 V. The observed identical (within error margins) transient properties of our OEGFETs will be beneficial when we integrate our sensors with peripheral electronics.

To demonstrate that our findings are consistent with our previously published works, we compared the measured capacitance data in the low frequency range (40–45 Hz), for each cortisol dilution. We observed a 10% increase in measured device capacitance with each step decrease in concentration, over the broad cortisol concentration range tested. This observation confirms that the correlation between gate capacitance and cortisol concentration is indeed present and drives the sensing mechanism of the OEGFET biosensor device.

## 5. Conclusions

Many novel EGFETs struggle to make the transition from laboratory prototypes to useable commercial devices due to their unpredictable electrical properties in multiplexed systems. We have proposed a simple parameter extraction method for predicting transient electrical characteristics of novel EGFETs to fill this vacant niche. The reported electrical characterization method was implemented on the analogous capacitor stacks of bio-gel EDLCs, and two commonly implemented EGFET configurations, OEGFETs and EGOFETs. A significant advantage of our equivalent circuit modeling approach is that it does not rely on mapping device action to conventional capacitor governing equations, rendering it highly adaptable to new experimental EGFET configurations. Our method reproduces the electrical input/output behavior of these novel capacitor stacks without relying on knowledge of internal capacitance parameters, an approach accepted and proven with conventional solid-state DLCs. Our process demonstrates a low error of <5% in reproducing the electrical characteristics of the bio-gel EDLC and the EGOFET equivalent, error low enough to be within tolerance limits for conventional capacitors, despite the vastly different τ and maximum charge capacity observed between these two device architectures.

The OEGFET equivalent parameter extraction method could reproduce the millisecond risetimes and discharges with 3% error between the simulated and real data. This is a significant finding, as effective simulation of the OEGFET equivalent translates to capturing the transient behavior in the label-free OEGFET biosensor capacitive stack. The OEGFET equivalent model demonstrated a measured 10% decrease in capacitance for each 10-fold increase in cortisol concentration. We have demonstrated the feasibility of electrically simulating these devices, thereby promoting the continued development of such promising devices into data nodes in commercially viable integrated devices.

This is the first parameter extraction method tailored specifically for organic bio-electrolytic devices, a necessary further improvement in device parameterization, and for the future implementations of these EDLC devices as biosensors and bioelectronic circuit components.
